# Computational Analysis of Naturally Occurring Aristolochic Acid Analogues and Their Biological Sources

**DOI:** 10.3390/biom11091344

**Published:** 2021-09-11

**Authors:** Tingjun Xu, Weiming Chen, Junhong Zhou, Jingfang Dai, Yingyong Li, Yingli Zhao

**Affiliations:** Shanghai Institute of Organic Chemistry, Chinese Academy of Sciences, 345 LingLing Road, Shanghai 200032, China; chenwm@sioc.ac.cn (W.C.); zhoujh8@sioc.ac.cn (J.Z.); daijf@sioc.ac.cn (J.D.); liyingyong@sioc.ac.cn (Y.L.); zhaoyl@sioc.ac.cn (Y.Z.)

**Keywords:** aristolochic acid analogues, biological sources, computational analysis, carcinogenic substance, computer predicted toxicity, natural products, toxic herbals, traditional medicines, virtual screening

## Abstract

Aristolochic acids are known for nephrotoxicity, and implicated in multiple cancer types such as hepatocellular carcinomas demonstrated by recent studies. Natural products that are analogues to aristolochic acids have been constantly isolated from organisms; a larger chemical space of these compounds and a wider coverage of biological sources should be determined in consideration of the potential hazard of aristolochic acid analogues and the wide distribution of their biological sources in the nature. Therefore, we carried out an in silico research of naturally occurring aristolochic acid analogues and their biological sources, as a supplement to existing studies. The result shows a chemical space of 238 naturally occurring aristolochic acid analogues that are present in 175 species of biological sources including 44 traditional medicines. With the computational estimation for toxicity and the implication in hazard assessment of a biological source with the presence of aristolochic acid analogues, we propose that additional awareness should be raised to the public for avoidance of toxic species, especially those that are used as herbal medicines and easily accessible.

## 1. Introduction

Human beings have a long history of taking herbaceous plants as medicines, and there is no doubt that the natural compounds derived from these biological sources are the treasure of potential drug candidates [1,2,3]. Nevertheless, no one can afford to neglect the dark side of an herbal medicine. Take the genus *Aristolochia*, for instance: Species of *Aristolochia* have been used for centuries as Traditional Chinese Medicine (TCM) in Asian countries and as herbal medicines in many other parts of the world [4]. But until the early 1990s, a weight loss treatment with *Aristolochia fangchi* (TCM name: Guang Fang Ji) at a Belgian clinic caused kidney failure, and then the medical event drew the attention of people for this toxic herbal medicine [5,6,7]. Epidemiological studies showed that the aristolochic acids (aristolochic acid I and aristolochic acid II) contained in *Aristolochia* are responsible for a high risk of nephrotoxicity and upper urinary tract carcinoma [8,9,10]. Furthermore, Ng et al. demonstrated that the aristolochic acids and their derivatives are widely implicated in hepatocellular carcinoma [11].

In the mechanism insights into the nephrotoxicity and carcinogenicity of aristolochic acids, a number of metabolites are further metabolized to aristolactams, which can be bio-activated by cytosolic and microsomal enzymes, and cause apoptosis in human proximal tubular cells and porcine renal tubular cells [12,13,14]. On the other hand, aristolochic acids-derived DNA adducts showed distinctive mutational signature, and cased the mutations in known cancer driver genes [11,15,16,17]. Aristolochic acid may be one of the strongest known mutagenic natural products on the human genome, comparing with mutation rates of smoking-associated lung cancer and UV radiation-associated melanoma [16,18,19]. Therefore, definite genotoxic and mutagenic mechanisms are involved in the medicinal plants that contain aristolochic acids.

In view of the above-mentioned toxicity showed by aristolochic acids and their derivatives, characteristics of molecular structure, which, similar to aristolochic acids, should be taken into account for reported nephrotoxicity and carcinogenicity, and the additional chemical space of the natural compounds, are classified as Aristolochic Acid Analogues (AAAs). The known AAAs primarily exist in the genera *Aristolochia* and *Asarum* [20,21], and, recently, more undocumented AAAs have been isolated from natural sources [22,23,24]. The suspected toxicity of biological products that contain AAAs deserves further investigation to reassure consumers that these products are safe, as discussed in a recent research by Ang P et al. [25]. However, there is no specific ban of medicinal and edible herbs containing AAAs in certain countries, especially those that are not included in Aristolochiaceae family and easily accessible; for example, the plant *Houttuynia cordata* (Chinese common name: Yu Xing Cao or Zhe Er Gen) is still widely used in China as potherb and even raw material of TCM injections [26,27]. Therefore, we suppose that there are more toxic natural products that should be classified as AAAs beyond the existing studies, and the AAAs widely exist in some species which have not attracted considerable attention. We herein describe a computational approach of delving deeper into AAAs, seeking out sufficient naturally occurring AAAs by virtual screening, and clarifying the relationships between AAAs and their biological sources, to find out the implicit chemical space of undocumented AAAs and the wide coverage of organisms that contain AAAs.

The approach of structure-based virtual screening is applicable for the computational task of searching and discovering exceptional molecules in a chemical database [28,29], and the targets of virtual screening implemented in this work could be naturally occurring AAAs. In the theoretical base of the used virtual screening, the conception is finding out the specific features of reported natural molecules that are categorized as AAAs, to determine which chemical structure is an analogue to aristolochic acids. In Structure Activity Relationship (SAR) or Quantitative Structure Activity Relationship (QSAR) studies, Molecular Similarity (MS) has been used to measure the similarity between molecular structures [30,31]. Calculations of MS based on substructures may be more suitable in this study than those based on molecular descriptors; in consideration of these concepts, some studies compare the scaffold of different compounds to determine the MS values and propose the similarity of biological activity by eliminating R-substituents [32,33,34]. Scaffold structure-based methods show better performance in obtaining structures with the same biological activity and finding out similar compounds among different families during the screening process [35]. Thus, the Maximum Common Substructure (MCS) of reported aristolochic acids can make the representation of the main features of AAAs.

Obtaining sufficient biological source information of a natural product used to be a profound investigation, given that the same natural product and its homologues can originate from various biological sources reported by multiple biomolecule-extracting research projects [36]. Thanks to the data resources of biological and chemical information, there is no need to peruse all of the related publications to search biological sources of a specific natural product. Although a large number of AAAs may exist in species of *Aristolochia* and other related species, their in vivo toxicities are largely unknown [21]. Computational toxicology estimations are intended to be used as part of a weight of evidence approach for hazard and risk assessment of AAAs, when there is an absent of laboratory experimental data [37].

## 2. Materials and Methods

### 2.1. Virtual Screening of Naturally Occurring Aristolochic Acid Analogues

**Figure 1:**
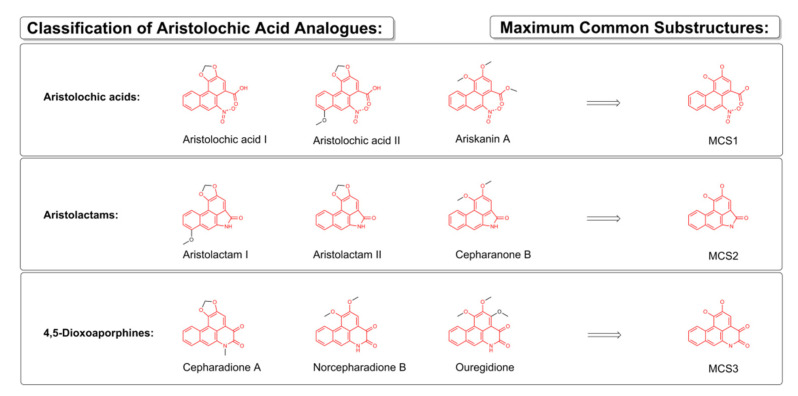
The classification and maximum common substructures of aristolochic acid analogues.

For virtual screening of AAAs, we first aimed at obtaining the largest common subgraph among a series of compounds that reported to be aristolochic acid analogues, as the MCSs of three categories of AAAs, as shown in Figure 1 (MCS1, MCS2 and MCS3). The category presenting the MSCs contains 109 compounds that reported to be analogues, curated from studies of aristolochic acids. Therefore, the identified MSCs may be distinctive from those that drew from other common datasets, as the compounds in this category are specialized in characteristics of molecular structure of AAAs. Then, we realized such an approach of virtual screening AAAs by calculating which chemical structure contains the MCSs; in the meantime, classification of AAAs would be determined if the input structure of a compound contained MCS1 (aristolochic acids), MCS2 (aristolactams) or MCS3 (4,5-dioxoaporphines).

The approach of virtual screening was implemented using RDKit library (version 2020.09.1) in Python (version 3.7). Three MCSs of the reported AAAs were achieved by using “Find MCS” function of RDKit library; default options for the algorithm were used to make sure that the approach exhaustively searches for a maximum common substructure from some time-out comparisons.

In order to make the coverage of this work aim at biological organism-derived compounds, as so-called “naturally occurring”, a natural product virtual library NPBS database was introduced in the used virtual screening method [36]. We imported all of the structures of natural products in NPBS and matched throughout with the MCSs described above to determine if the target natural product molecule contains maximum common substructure of reported AAAs. The structural matching algorithm of comparing target natural product molecules and the MCSs was achieved by “Substructure Searching” function of RDKit library.

### 2.2. Obtaining Information Data of Biological Sources Containing Aristolochic Acid Analogues

**Figure 2:**
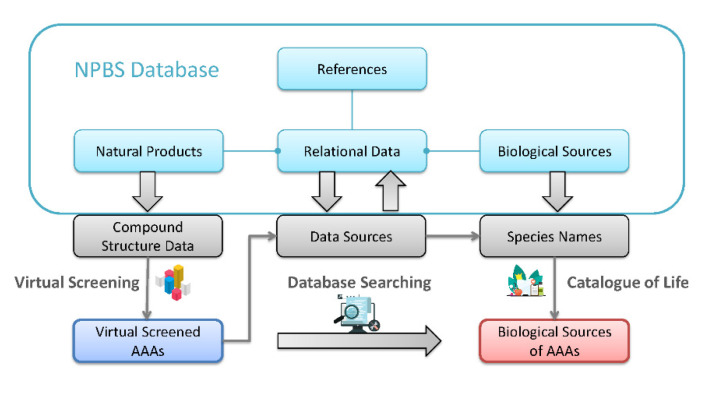
The process flow diagram of obtaining information data of biological sources containing aristolochic acid analogues.

As we screened out AAAs from natural product data collection of NPBS by the approach described above, the biological source information of AAAs can be drawn from this extraordinary data resource, as shown in Figure 2.

The biological source information we obtained from NPBS is the species name of experimental materials described in biomolecule-extracting research articles [36]. The published scientific names may be revised by taxonomists over time, and the species names may be synonyms in taxonomy. Thus, we resorted to Catalogue of Life (COL), the international community for listing species, for seeking the authentic biological sources of organisms that contain AAAs. In addition, the synonymies’ species names can be accessed to the accepted species name, which are referenced by any scientific names described in various research articles.

We used the reference information (Supplementary Data 5) for the primary literatures that reported the relational data between AAAs and their biological sources in order to validate the result data of species names and chemical structures.

### 2.3. Computational Toxicology Estimations of Aristolochic Acid Analogues

Computer-predicted toxicity values of AAAs were calculated using the ACD/Percepta software (Version 2020, Advanced Chemistry Development Inc.) with modules of “Acute Toxicity”, “Genotoxicity” and “Health Effects”. We implemented interactive and responsive charting analysis of the result data by using G2Plot library (version 2.3.12) in Python (version 3.7).

## 3. Results

### 3.1. Chemical Space of Naturally Occurring Aristolochic Acid Analogues

**Figure 3:**
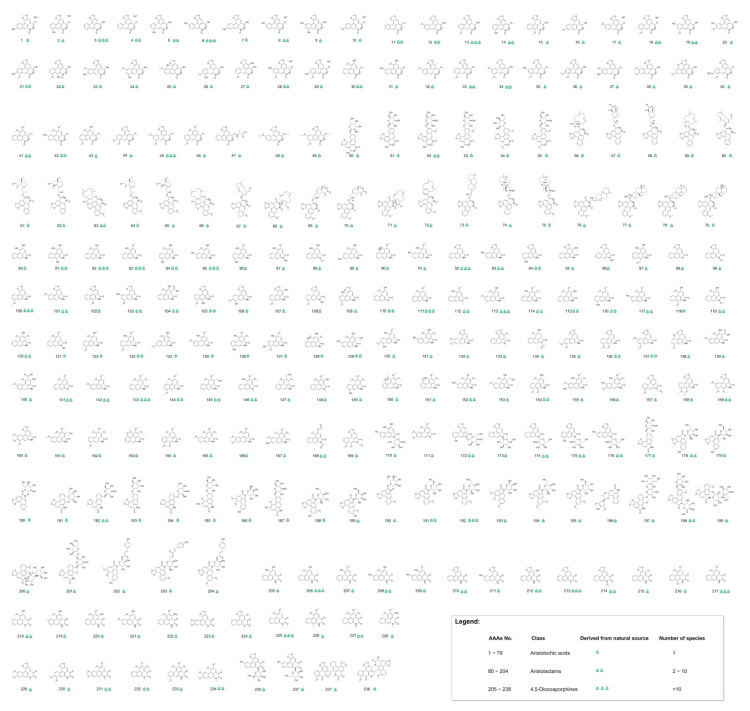
The result of naturally occurring aristolochic acid analogues obtained by virtual screening. High-resolution image file of this figure can be found in Supplementary Materials as Figure S1.

Comprehensive classification of AAAs is based on molecular structural features of reported AAAs, and three categories of AAAs are classified as aristolochic acids, aristolactams and 4,5-dioxoaporphines. The used approach of virtual screening in this work resulted in 238 naturally occurring AAAs (the serial number of aristolochic acid analogues listed under each structure is defined as “AAAs No.”), including 79 aristolochic acids (AAAs No. 1–79), 125 aristolactams (AAAs No. 80–204) and 34 4,5-dioxoaporphines (AAAs No. 205–238). The AAAs are all reported as natural products and having specific species of biological sources; 80 AAAs are present in more than 2 species, and 17 AAAs are present in more than 10 species (Supplementary Data 1), as shown in Figure 3.

**Figure 4:**
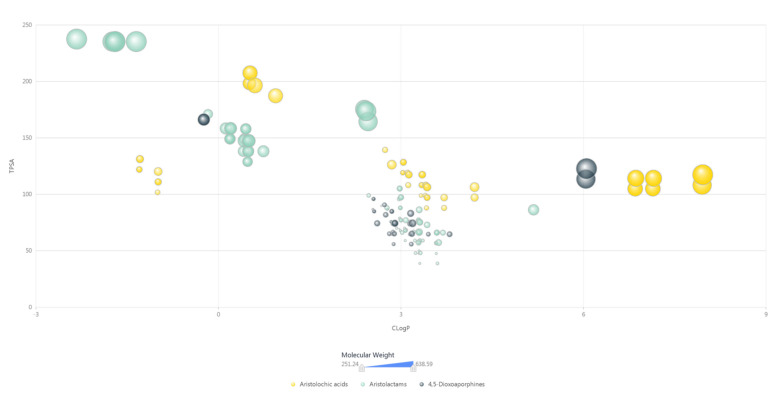
The scatter plot of molecular properties of aristolochic acid analogues. High-resolution image file of this figure can be found in Supplementary Materials as Figure S2.

As observed from computed molecular properties data (Supplementary Data 2), the molecules of AAAs consist of at least 3 and up to 15 oxygen atoms and at least 1 nitrogen atom and have an average molecular weight value of 381.24. The Calculated LogP (CLogP) and Topological Polar Surface Area (TPSA) values of AAAs scatter in the range of −2.33–7.96 and 38.77–237.53, as showed in Figure 4. More detailed computable molecular properties including computer-predicted physicochemical properties are presented in Supplementary Data 3 to show the further chemical space of AAAs.

### 3.2. Coverage of Biological Sources Containing Aristolochic Acid Analogues

**Figure 5:**
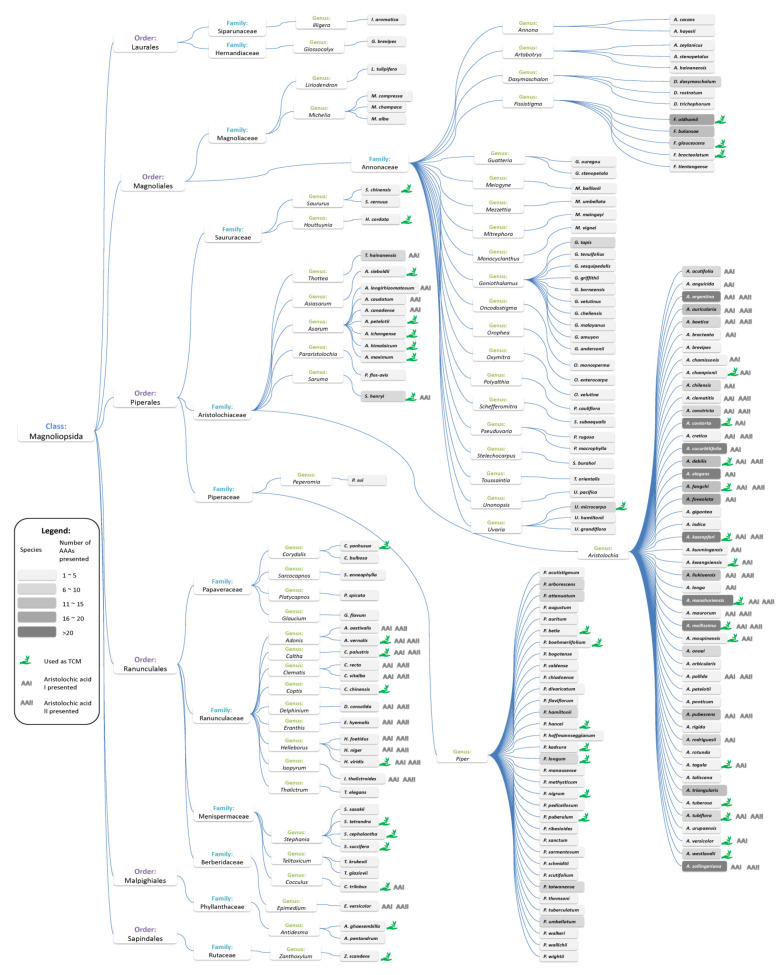
The taxonomic distribution of species containing aristolochic acid analogues. High-resolution image file of this Figure can be found in Supplementary Materials as Figure S3.

A total of 175 species of biological sources (Supplementary Data 4) are revealed to contain AAAs. The biological sources include not only species of Aristolochiaceae but various plants in 13 families of the class Magnoliopsida (Figure 5). The biological sources are reported to contain at least 1 AAAs, 22 of them containing more than 10 AAAs, and the species of *Aristolochia kaempferi* is reported to contain the maximum number of AAAs. Aristolochic acid I (AAAs No. 13) and aristolochic acid II (AAAs No. 3), which are considered to be the main constituents responsible for the nephrotoxic and carcinogenic effects, are present in 52 species; it is particularly remarkable that these two compounds are widespread in the species of Aristolochiaceae and Ranunculaceae. According to our investigation (Supplementary Data 4), 44 species of the biological sources have been used as TCM, including *Aristolochia kaempferi* (TCM name: Han Zhong Fang Ji), *Aristolochia contorta* (TCM name: Ma Dou Pu), *Aristolochia manshuriensis* (TCM name: Guan Mu Tong), and *Aristolochia mollissima* (TCM name: Xun Gu Feng), which not only contain more than 20 AAAs, but also are reported to contain both aristolochic acid I and aristolochic acid II. More detailed information of the biological sources including scientific, synonymous, common, and TCM names are presented in Supplementary Data 4 to show the further coverage of biological sources containing AAAs.

### 3.3. Relationship of Aristolochic Acid Analogues and Their Biological Sources

**Figure 6:**
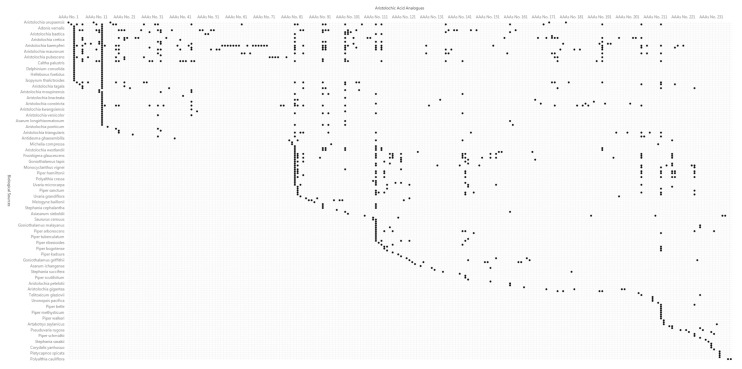
Relationship matrix analysis of aristolochic acid analogues and their biological sources. High-resolution image file of this figure can be found in Supplementary Materials as Figure S4.

In the relationship matrix analysis (Figure 6) of AAAs and their biological sources, we found that the same AAAs can originate from various species, which may be irrelevant in biological taxonomy, and the three categories of AAAs (aristolochic acids, aristolactams and 4,5-dioxoaporphines) may coexist in a specific species of biological sources. The genus *Aristolochia* covers the most diverse of AAAs, which may be a significant factor of toxicity of the plants belonging to this genus. The table of relational data (Supplementary Data 5) between AAAs and their biological sources was achieved from this work in order to see which species of biological source contain a specific AAAs based on column “AAAs No.” and the table of AAAs data (Supplementary Data 1), for example, the analysis of species containing aristolochic acid I (AAAs No. 13). Similarly, AAAs that are present in a specific species can be found based on column “Biological Sources” and the table of biological source data (Supplementary Data 4), for example, the analysis of AAAs that are present in the species of *Aristolochia kaempferi* or the TCM “Han Zhong Fang Ji”. Moreover, all of the 781 records of relational data are accompanied with references, from which the AAAs were reported.

### 3.4. Computer Predicted Toxicity of Aristolochic Acid Analogues

**Figure 7:**
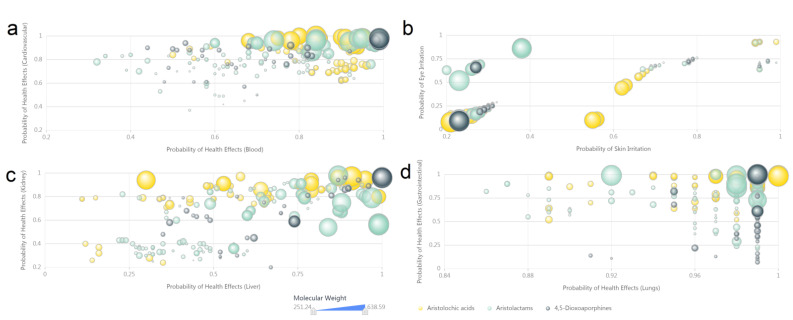
The scatter plot of computer-predicted health effects of aristolochic acid analogues. (**a**) Probability of health effects (blood and cardiovascular). (**b**) Probability of irritation (skin and eye). (**c**) Probability of health effects (liver and kidney). (**d**) Probability of health effects (lungs and gastrointestinal). High-resolution image file of this figure can be found in Supplementary Materials as Figure S5.

**Figure 8:**
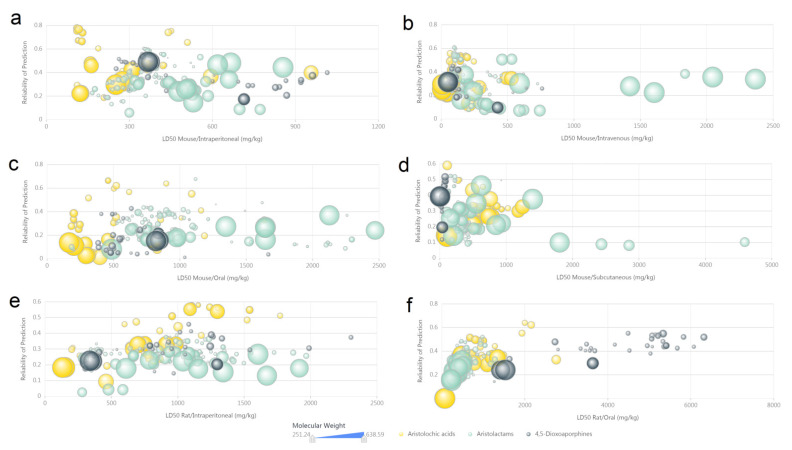
The scatter plot of computer predicted acute toxicity of aristolochic acid analogues. (**a**) LD50 mouse/intraperitoneal (mg/kg). (**b**) LD50 mouse/intravenous (mg/kg). (**c**) LD50 mouse/oral (mg/kg). (**d**) LD50 mouse/subcutaneous (mg/kg). (**e**) LD50 rat/intraperitoneal (mg/kg). (**f**) LD50 rat/oral (mg/kg). High-resolution image file of this figure can be found in Supplementary Materials as Figure S6.

**Figure 9:**
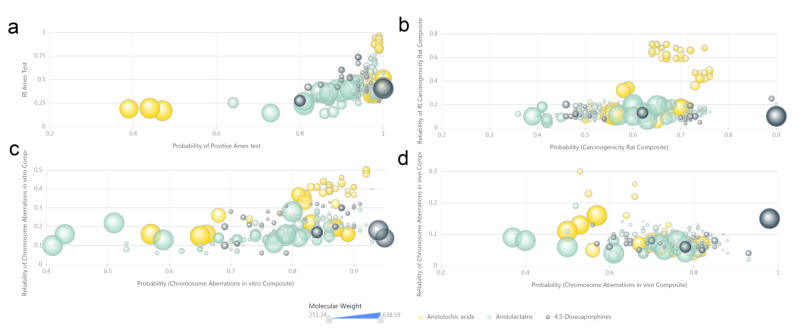
The scatter plot of computer predicted genotoxicity of aristolochic acid analogues. (**a**) Probability of positive Ames test. (**b**) Probability of carcinogenicity rodent composite. (**c**) Probability of chromosome aberrations in vitro composite. (**d**) Probability of chromosome aberrations in vivo composite. High-resolution image file of this figure can be found in Supplementary Materials as Figure S7.

Charting analysis results of the computer-predicted toxicity data (Supplementary Data 6) of AAAs are shown in three categories: health effects (Figure 7), acute toxicity (Figure 8) and genotoxicity (Figure 9). Most of AAAs exhibit serious possibility of health effects according to the calculated values, especially in consistency of performance effects on blood (Figure 7a, *x*-axis), cardiovascular (Figure 7a, *y*-axis), liver (Figure 7c, *x*-axis), kidney (Figure 7c, *y*-axis) and lungs (Figure 7d, *x*-axis). AAAs containing 2-aminoethanol fragment or carboxyl without basic groups are more likely to irritate eyes (Figure 7b, *x*-axis) and skin (Figure 7b, *y*-axis).

In the analysis of computer-predicted acute toxicity, we found that aristolochic acids may be more toxic than aristolactams and 4,5-dioxoaporphines, in view of the lethal dose distribution of mouse intraperitoneal values (Figure 8a), mouse oral values (Figure 8c) and rat intraperitoneal values (Figure 8e). Despite moderate reliability of the calculation, AAAs exhibit hypertoxic potential as observed from computer-predicted acute toxicity data, especially in the lethal dose of mouse intravenous values (Figure 8b), mouse subcutaneous values (Figure 8d) and rat oral values (Figure 8f).

The data of computer-predicted genotoxicity exhibit considerable mutagenic probability of AAAs, seeing the high probability values in positive Ames test (Figure 9a), carcinogenicity rodent composite (Figure 9b) and chromosome aberrations in vitro (Figure 9c) and in vivo composite (Figure 9d). Specifically, the vast majority of AAAs have more than 0.8 probability of positive Ames test (Figure 9a), and the predicted values of aristolochic acids seem to be more reliable than aristolactams and 4,5-dioxoaporphines (Figure 9b,c). More detailed computer-predicted toxicity data including probability values of cytochrome P450 inhibitors and human ether-a-go-go (hERG) channel inhibitors are presented in Supplementary Data 6 to show the further hazardous potential of AAAs.

### 3.5. Exclusive Aristolochic Acid Analogues

**Figure 10:**
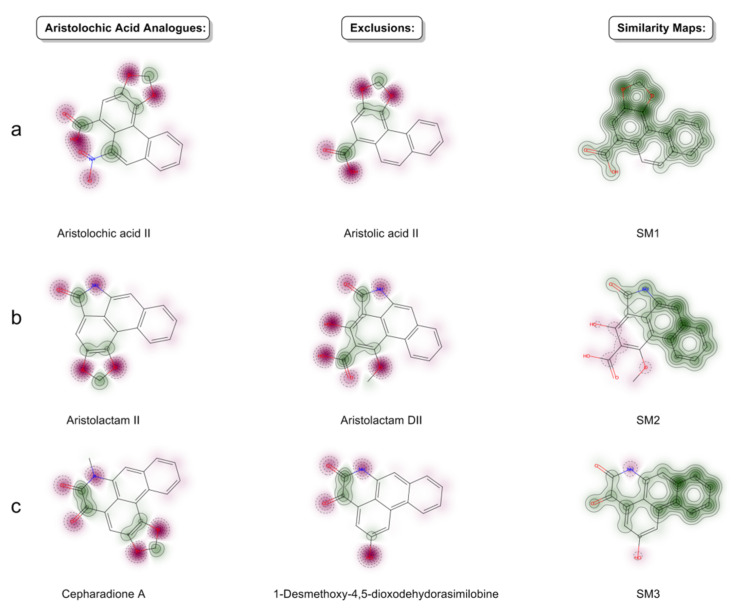
Analysis of Gasteiger partial charges and similarity maps between aristolochic acid analogues and exclusions. (**a**) An example of aristolochic acids (aristolochic acid II) and exclusive AAAs (aristolic acid II). (**b**) An example of aristolactams (aristolactam II) and exclusive AAAs (aristolactam DII). (**c**) An example of 4,5-dioxoaporphines (cepharadione A) and exclusive AAAs (1-desmethoxy-4,5-dioxodehydorasimilobine).

Although reported as aristolochic acid analogues, 17 natural products mainly derived from the genus *Aristolochia* are classified as exclusions of AAAs from this work. Analysis of Gasteiger partial charges and Similarity Maps (SM) between the AAAs and the exclusions are visualized in Figure 10. The chemical difference between the three categories of AAAs and the compounds in Supplementary Data 7, particularly the absence of key groups such as nitro groups (Figure 10a) and methylenedioxyphenyls (Figure 10b,c), make the exclusions fail in matching with the structural features of compounds that can be categorized as AAAs in this work. The missing groups would make the exclusions behave largely differently from AAAs in bioactivity. Specifically, the nitro group, which is absent from aristolic acid II (Figure 10a), would exhibit genotoxic activity after metabolic transformation to hydroxylamines [38,39]. The result data of exclusive AAAs are contained in Supplementary Data 7 accompany with biological sources and references.

## 4. Discussion

From some reports, aristolochic acids have been detected in secondary metabolite of aposematic butterflies (*Battus polydamas*) [40,41,42]. It is believed that the aristolochic acids that occur in these *Aristolochia*-feeding butterflies are from their host plants rather than from manufacturing their own defensive substances [43]. Some of the aristolochic acids that are not present in *Aristolochia galeata* leaves occur in *Battus polydamas* larvae reared on these leaves; it is speculated that they may be synthesized by the larvae from chemical precursors in the plant [44]. However, these aristolochic acids are counted in the AAAs of this work, and are associated with biological sources of other species in the genus *Aristolochia*; thus, the possibility of alternative food source for *Battus polydamas* larvae should be considered.

Artifacts would arise from the products of non-enzymatic reactions (e.g., solvolysis and oxidation) during the process of natural compounds extraction or purification [45]. AAAs with carboxylic groups may have esterification with the common solvents of alcohols, and phenols may yield methides with nucleophilic solvents (e.g., methanol). For example, 4,5-dioxodehydroasimilobin (AAAs No. 206) may be an artifact of norcepharadione B (AAAs No. 217), based on previous findings [29]. Therefore, original forms of AAAs may exist in the organisms of their biological sources, and the additional cases of undocumented AAAs deserve further research.

A number of species in the biological sources (Supplementary Data 4) have not been reported for the presence of aristolochic acids, but were associated with various aristolactams and 4,5-dioxoaporphines in the result of this work (Supplementary Data 5), which are implicit in the metabolites of aristolochic acids and not banned in certain countries. Therefore, these species should also be aware of their toxicity, especially those that are widely used as herbal medicines and are easily accessible, such as *Fissistigma oldhamii* (TCM name: Guang Xiang Teng), *Saururus chinensis* (TCM name: San Bai Cao), *Asarum maximum* (TCM name: Da Xi Xin), and so on. What draws more of our attention is that some eatable species are involved in the biological sources containing AAAs; for example, roots of *Houttuynia cordata* (TCM name: Yu Xing Cao) are used as potherbs, fruits of *Uvaria microcarpa* (TCM name: Jiu Bing Po) are used for distillers yeast, leafs of *Antidesma ghaesembilla* (TCM name: Tian Bian Mu) are used for making tea, and quite a few species in the genus *Piper* are used as spices of peppers.

In the comparison of computer-predicted toxicity data and ground truth of experimental data, the result supported our hypothesis and confirmed the predictions. A great number of AAAs presented by this work have cytotoxicity when isolated from natural sources, according to the bioactivity results found by Reaxys for searching a substance of AAAs. Some of the AAAs exhibit considerable genotoxicity, according to related studies of in vitro toxicity tests of AAAs [46,47,48], which is consistent with our predictions (see Table S1).

Toxicology and safety assessment of an herbal medicine could be complicated and confused because it could contain substances both beneficial and noxious to human health, and there will be considerable accumulation of toxins resulting in toxic reaction. Even though an herbal medicine contains AAAs, it might be harmless if its quantity is negligible, considering the abundance of toxic substances. As observed from the results of this work (Figure 5 and Supplementary Data 4), plants containing diverse AAAs with unknown concentrations have also been demonstrated to be toxic. The findings here indicate that the hazard assessment of a biological source can show the presence of AAAs and the number of AAAs that can be used to assess the toxicity of this species, when there are not much in vivo toxicity studies available to the public. When ranking the traditional medicines based on the number of AAAs (Supplementary Data 4), we found that the top-ranked medicines are also reported by related research projects to be of high carcinogenic risks [49,50,51]. To the best of our understanding, more traditional medicines should be involved in light of the wide usage of AAA-containing plants in TCM formulations.

## 5. Conclusions

In silico toxicology estimation is not intended as a substitute for appropriate animal or clinical studies, and computer-predicted toxicity data of AAAs are not enough to determine the potential hazard of their biological sources. However, with the panorama of AAAs and their biological sources, there will be a toxicological profile of species containing AAAs. In view of the fact that the biological sources involve a considerable number of TCMs that can be easily available by online purchases, the result data of this study may act as warnings to herbal medicine abuse.

## Data Availability

The data presented in this study are available in Supplementary Materials.

## Supplementary Materials

The following are available online at https://www.mdpi.com/article/10.3390/biom11091344/s1, Figure S1: The result of naturally occurring aristolochic acid analogues obtained by virtual screening, Figure S2: The scatter plot of molecular properties of aristolochic acid analogues, Figure S3: The taxonomic distribution of species containing aristolochic acid analogues, Figure S4: Relationship matrix analysis of aristolochic acid analogues and their biological sources, Figure S5: The scatter plot of computer-predicted health effects of aristolochic acid analogues, Figure S6: The scatter plot of computer-predicted acute toxicity of aristolochic acid analogues, Figure S7: The scatter plot of computer-predicted genotoxicity of aristolochic acid analogues, Supplementary Data 1: Data list of aristolochic acid analogues, Supplementary Data 2: Molecular properties data of aristolochic acid analogues, Supplementary Data 3: Computer-predicted physicochemical properties of aristolochic acid analogues, Supplementary Data 4: Data list of biological sources, Supplementary Data 5: Relational data and references, Supplementary Data 6: Computer-predicted toxicity data of aristolochic acid analogues, Supplementary Data 7: Data list of exclusive aristolochic acid analogues, Supplementary Data 8: Codes for virtual screening, properties computing and data analyzing, Supplementary Data 9: Interactive and responsive charting analysis files, Supplementary Data 10: Table S1—Some of the experimental data of AAAs for supporting the prediction.

## Abbreviations

TCM	Traditional Chinese Medicine
AAAs	Aristolochic Acid Analogues
SAR	Structure Activity Relationship
QSAR	Quantitative Structure Activity Relationship
MS	Molecular Similarity
MCS	Maximum Common Substructure
NPBS	Natural Product and Biological Source database
COL	Catalogue of Life
