# Supplementary material for: Computational Analysis of Naturally Occurring Aristolochic Acid Analogues and Their Biological Sources
Source: Biomolecules. 2021 Sep 11;11(9):1344. doi: 10.3390/biom11091344 (PMC8471445; doi:10.3390/biom11091344)
Supplement: Supplementary file 1 [file biomolecules-11-01344-s001.zip › Supplementary Materials/Supplementary Data/Supplementary Data 9/Analysis of computer predicted acute toxicity_LD50 Mouse IP.html]

PyG2Plot
