# Supplementary material for: Computational Analysis of Naturally Occurring Aristolochic Acid Analogues and Their Biological Sources
Source: Biomolecules. 2021 Sep 11;11(9):1344. doi: 10.3390/biom11091344 (PMC8471445; doi:10.3390/biom11091344)
Supplement: Supplementary file 1 [file biomolecules-11-01344-s001.zip › Supplementary Materials/Supplementary Figures/Figure S1.pdf]

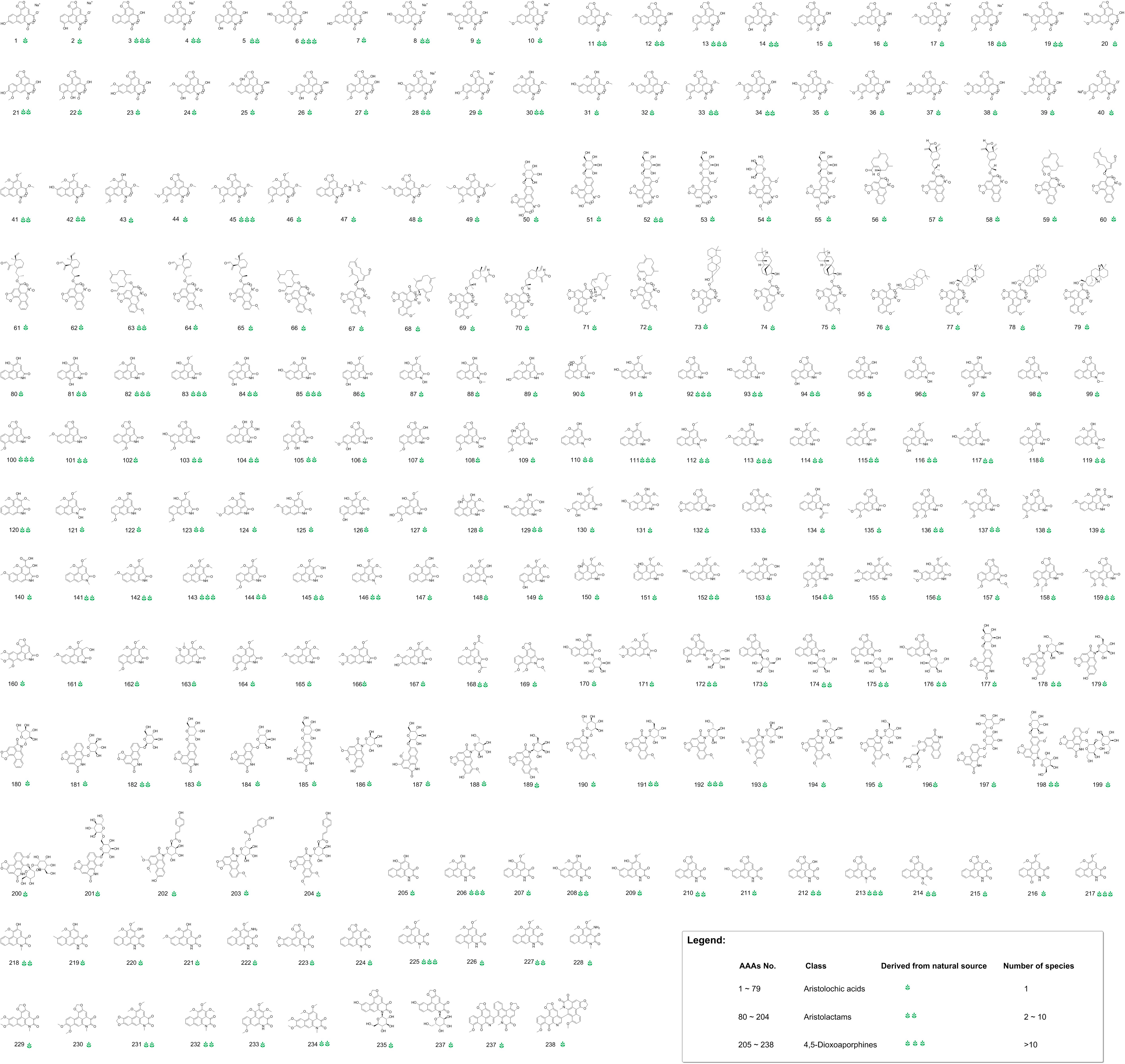
Legend:
AAAs No.
Class
Derived from natural source
Number of species
1 ~ 79
Aristolochic acids
1
80 ~ 204
Aristolactams
2 ~ 10
205 ~ 238
4,5-Dioxoaporphines
>10
