# Supplementary material for: Computational Analysis of Naturally Occurring Aristolochic Acid Analogues and Their Biological Sources
Source: Biomolecules. 2021 Sep 11;11(9):1344. doi: 10.3390/biom11091344 (PMC8471445; doi:10.3390/biom11091344)
Supplement: Supplementary file 1 [file biomolecules-11-01344-s001.zip › Supplementary Materials/Supplementary Figures/Figure S6.pdf]

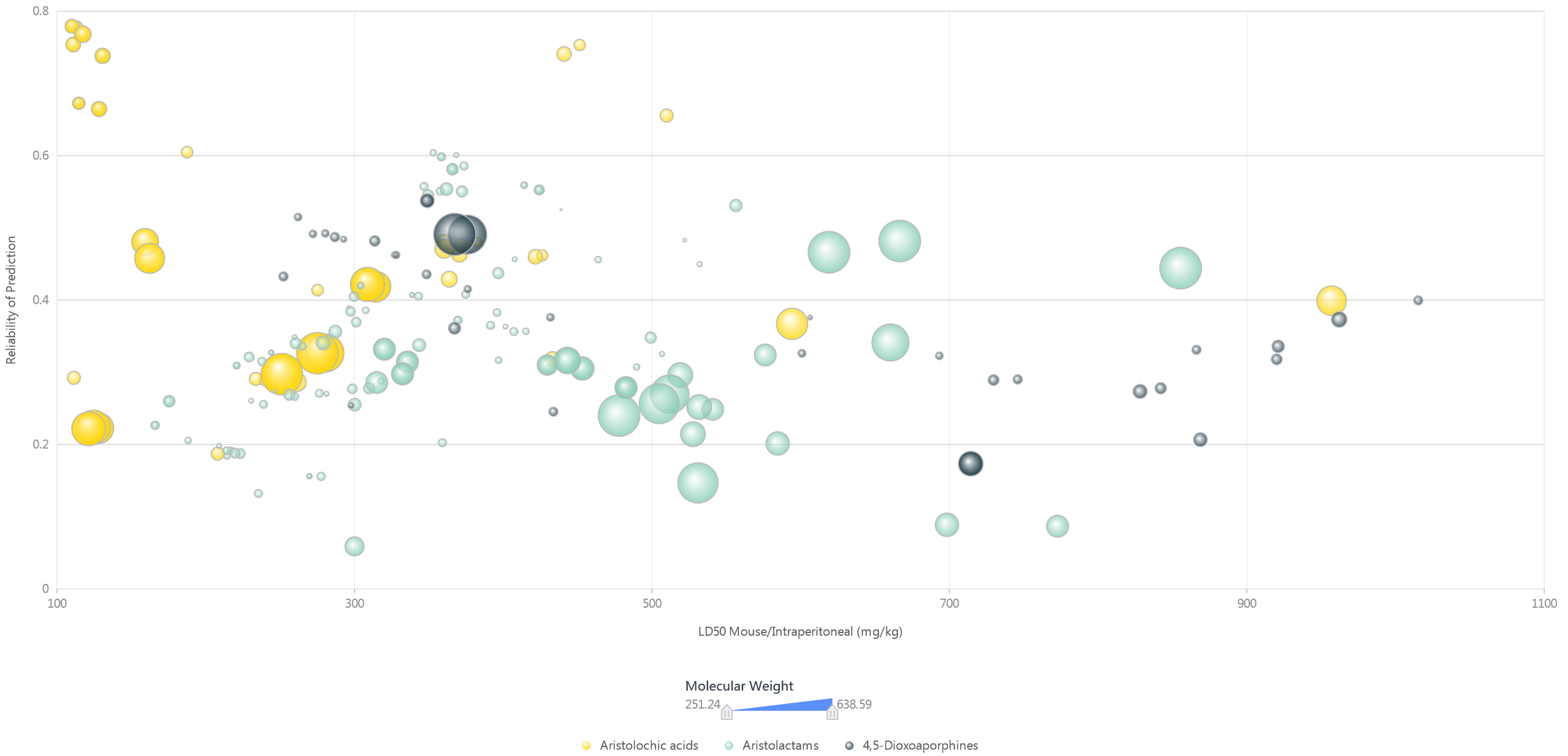

Reliability of Prediction
0.8
0.6
0.4
0.2
0
100
300
500
700
900
1100
LD50 Mouse/Intraperitoneal (mg/kg)
Molecular Weight
251.24
638.59
Aristolochic acids
Aristolactams
4,5-Dioxoaporphines

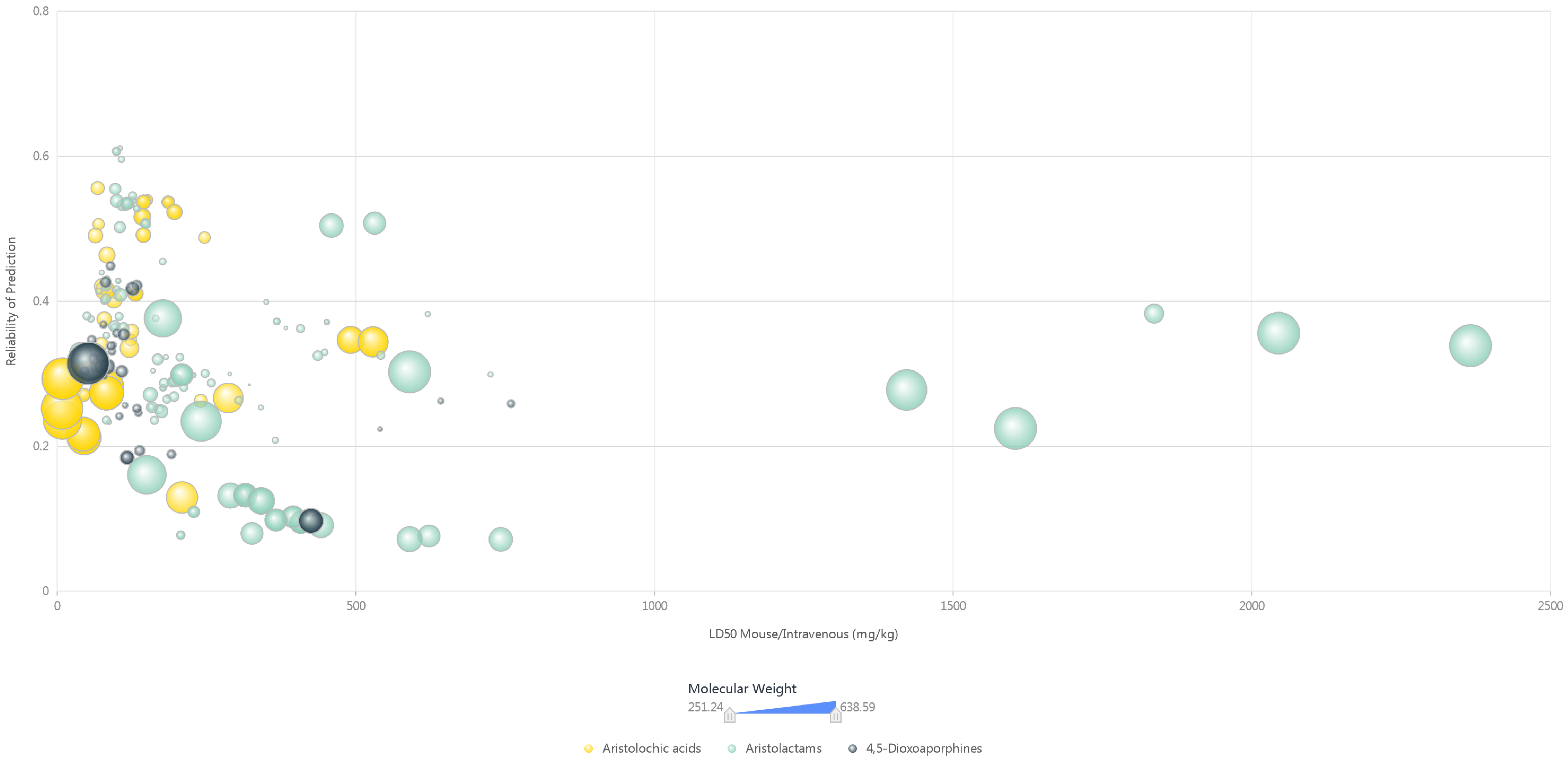

0.8
0.6
0.4
0.2
0
Reliability of Prediction
0
500
1000
1500
2000
2500
LD50 Mouse/Intravenous (mg/kg)
Molecular Weight
251.24
638.59
Aristolochic acids
Aristolactams
4,5-Dioxoaporphines

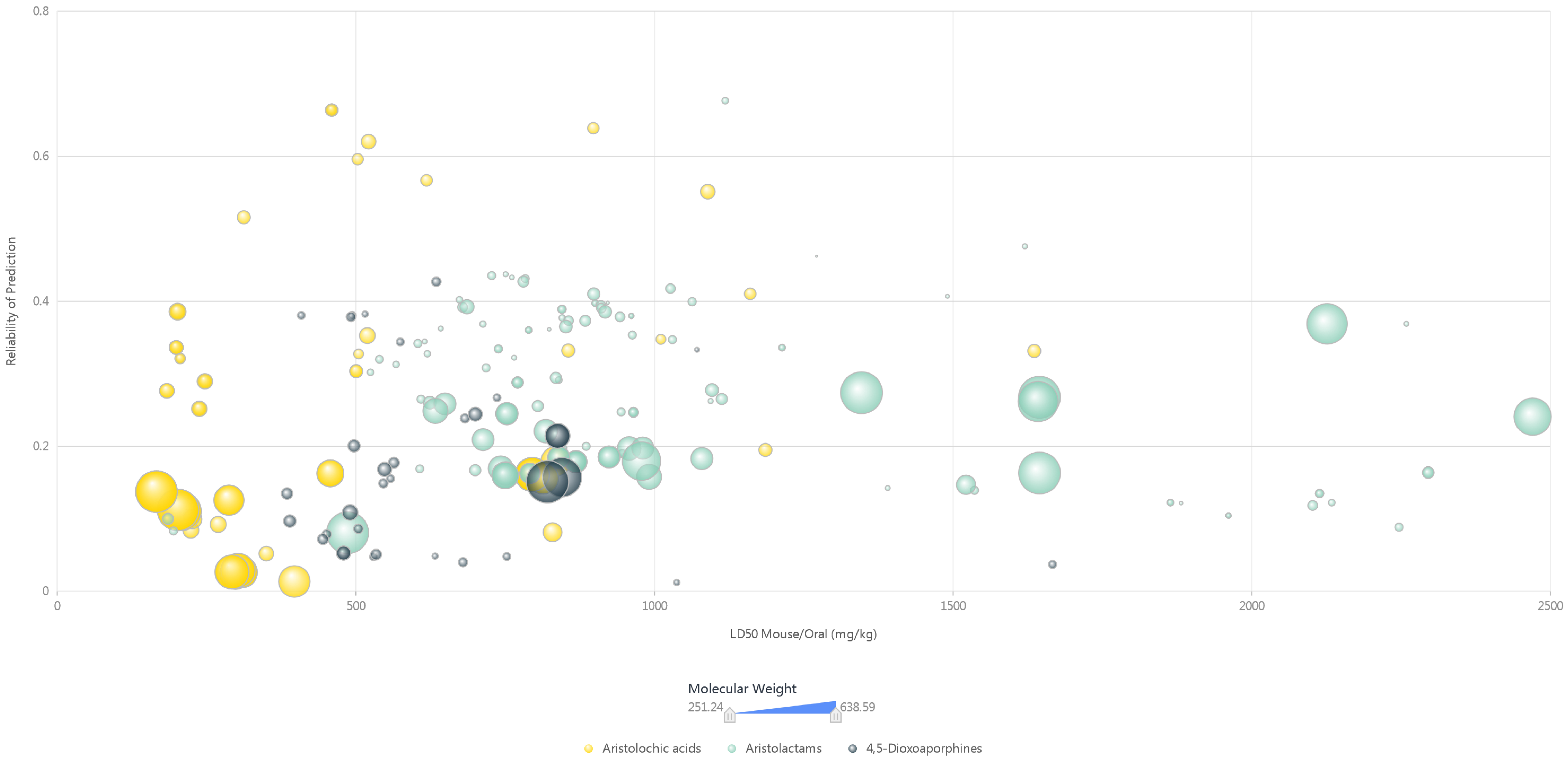

0.8
0.6
0.4
0.2
0
Reliability of Prediction
0
500
1000
1500
2000
2500
LD50 Mouse/Oral (mg/kg)
Molecular Weight
251.24
638.59
Aristolochic acids
Aristolactams
4,5-Dioxoaporphines

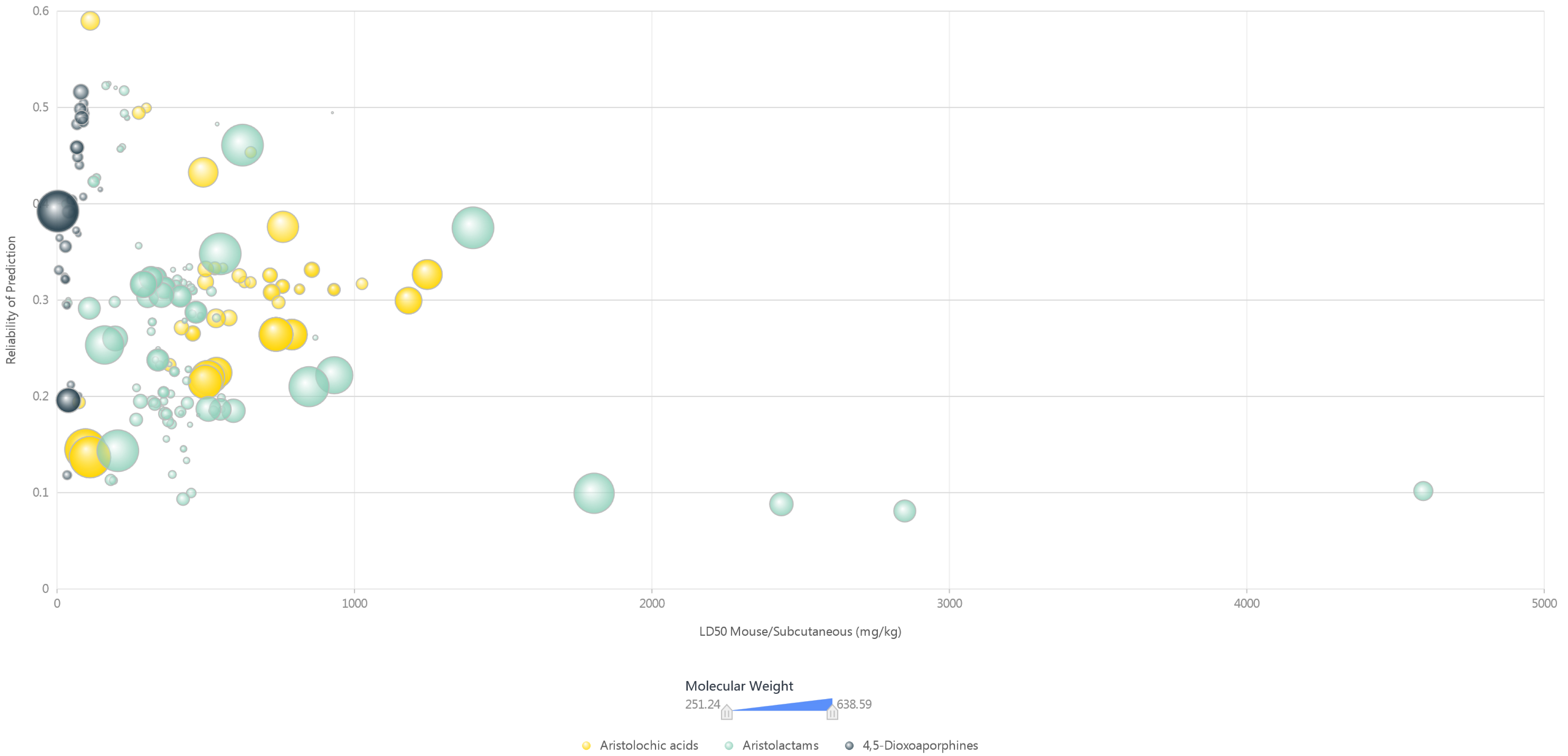

Reliability of Prediction
0.6
0.5
0.3
0.2
0.1
0
0
1000
2000
3000
4000
5000
LD50 Mouse/Subcutaneous (mg/kg)
Molecular Weight
251.24
638.59
Aristolochic acids
Aristolactams
4,5-Dioxoaporphines

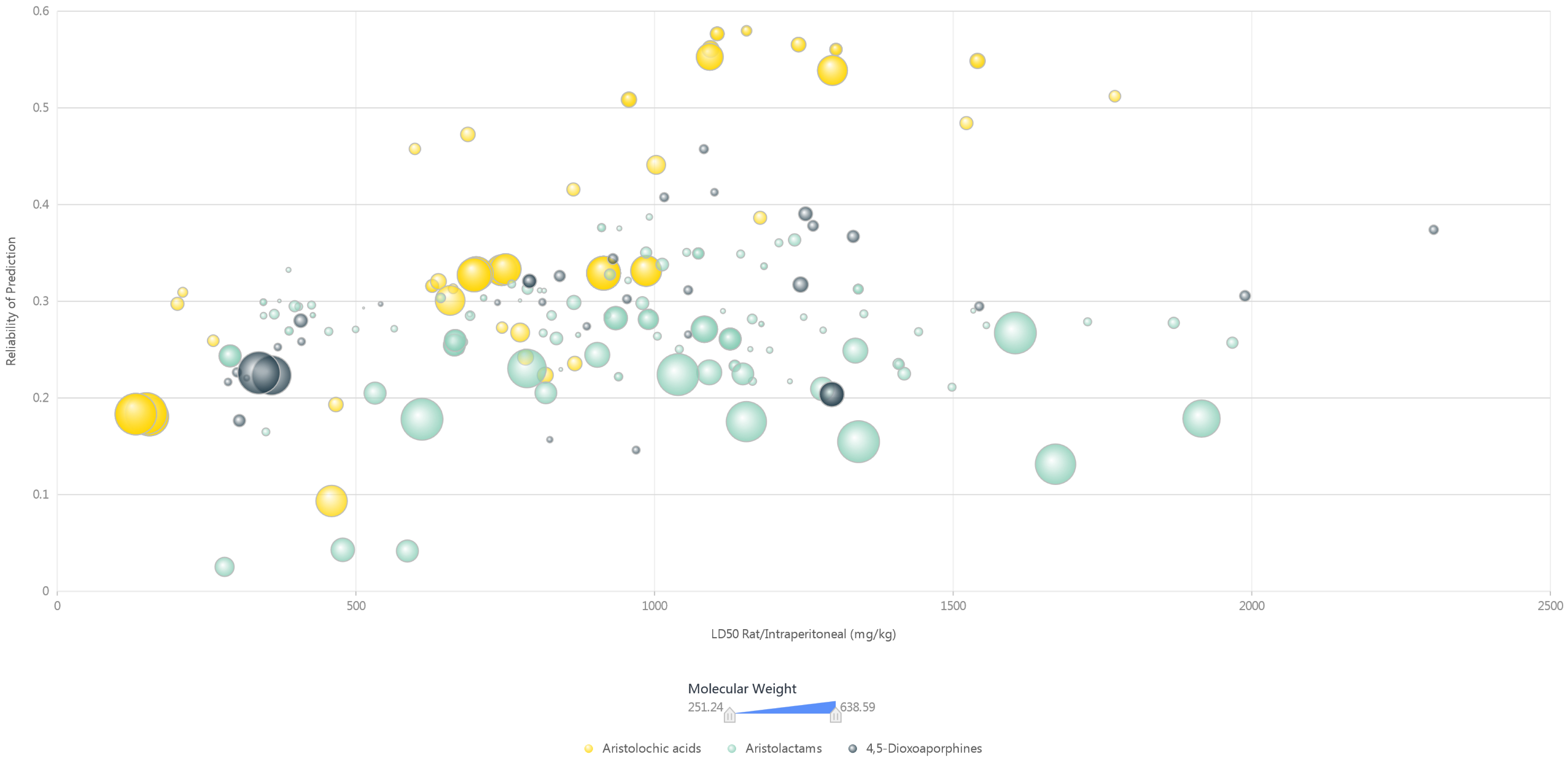
0.6
0.5
0.4
0.3
0.2
0.1
0
Reliability of Prediction
0
500
1000
1500
2000
2500
LD50 Rat/Intraperitoneal (mg/kg)
Molecular Weight
251.24
638.59
Aristolochic acids
Aristolactams
4,5-Dioxoaporphines

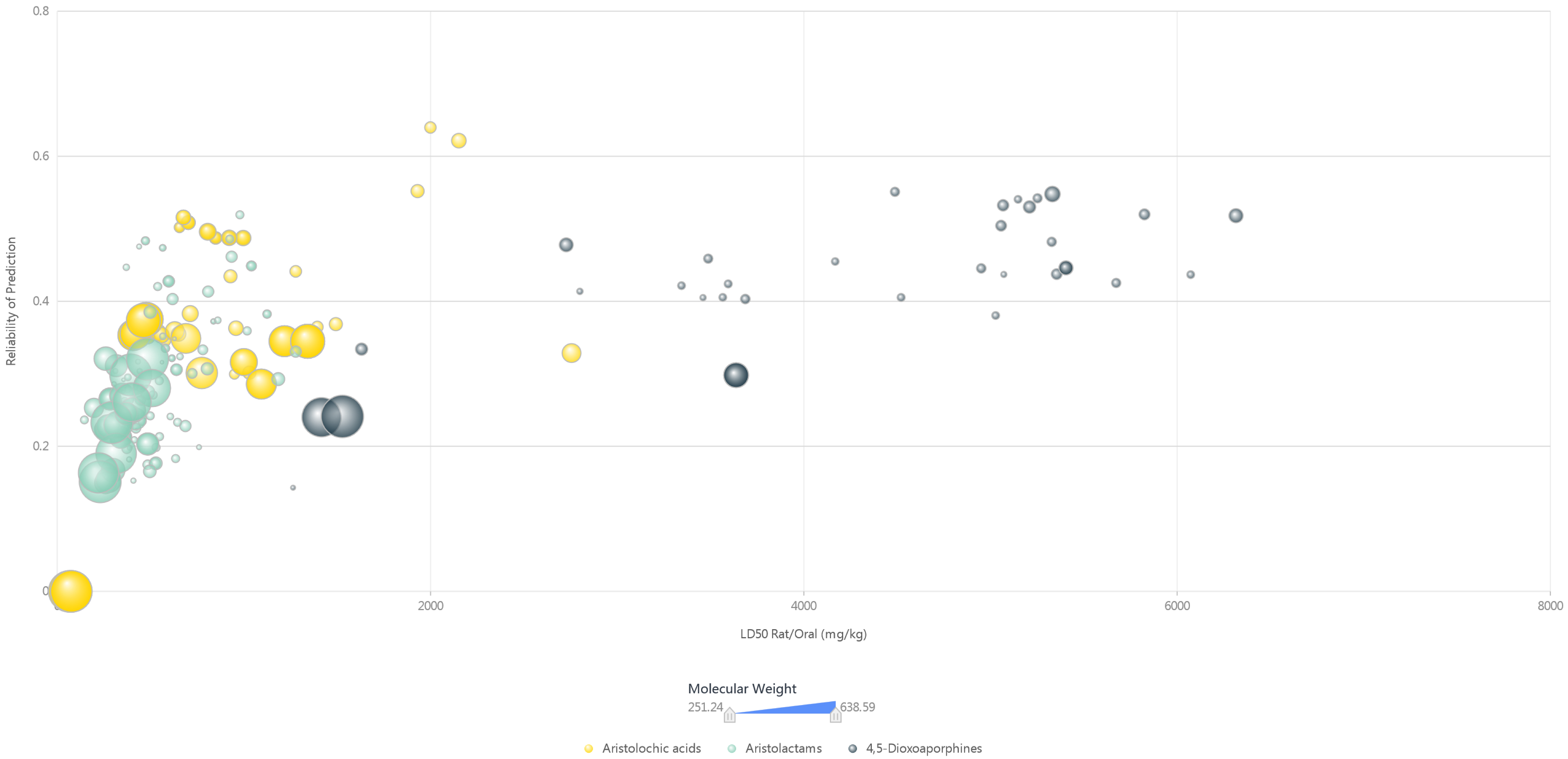

0.8
0.6
0.4
0.2
0
Reliability of Prediction
2000
4000
6000
8000
LD50 Rat/Oral (mg/kg)
Molecular Weight
251.24
638.59
Aristolochic acids
Aristolactams
4,5-Dioxoaporphines
