# Supplementary figures and images for: Computational Analysis of Naturally Occurring Aristolochic Acid Analogues and Their Biological Sources
Source: Biomolecules. 2021 Sep 11;11(9):1344. doi: 10.3390/biom11091344 (PMC8471445; doi:10.3390/biom11091344)

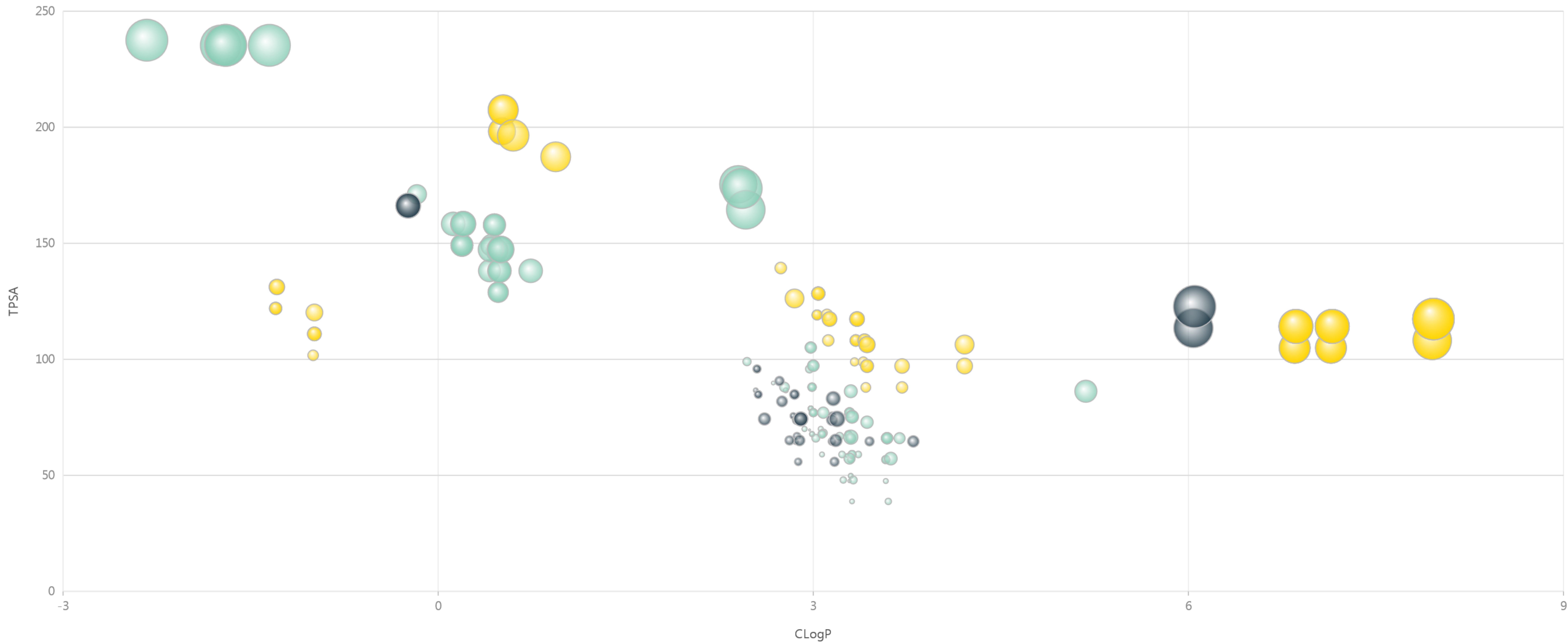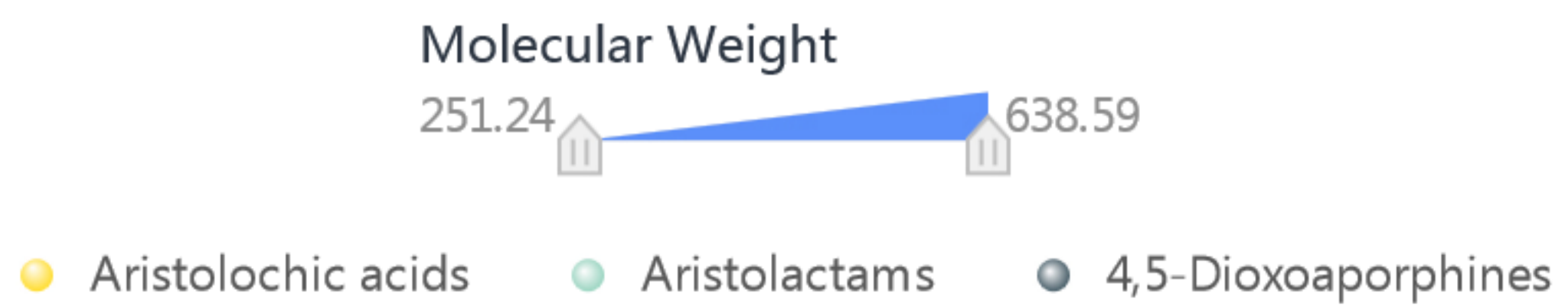

Supplement: Supplementary file 1 [file biomolecules-11-01344-s001.zip › Supplementary Materials/Supplementary Figures/Figure S2.pdf]

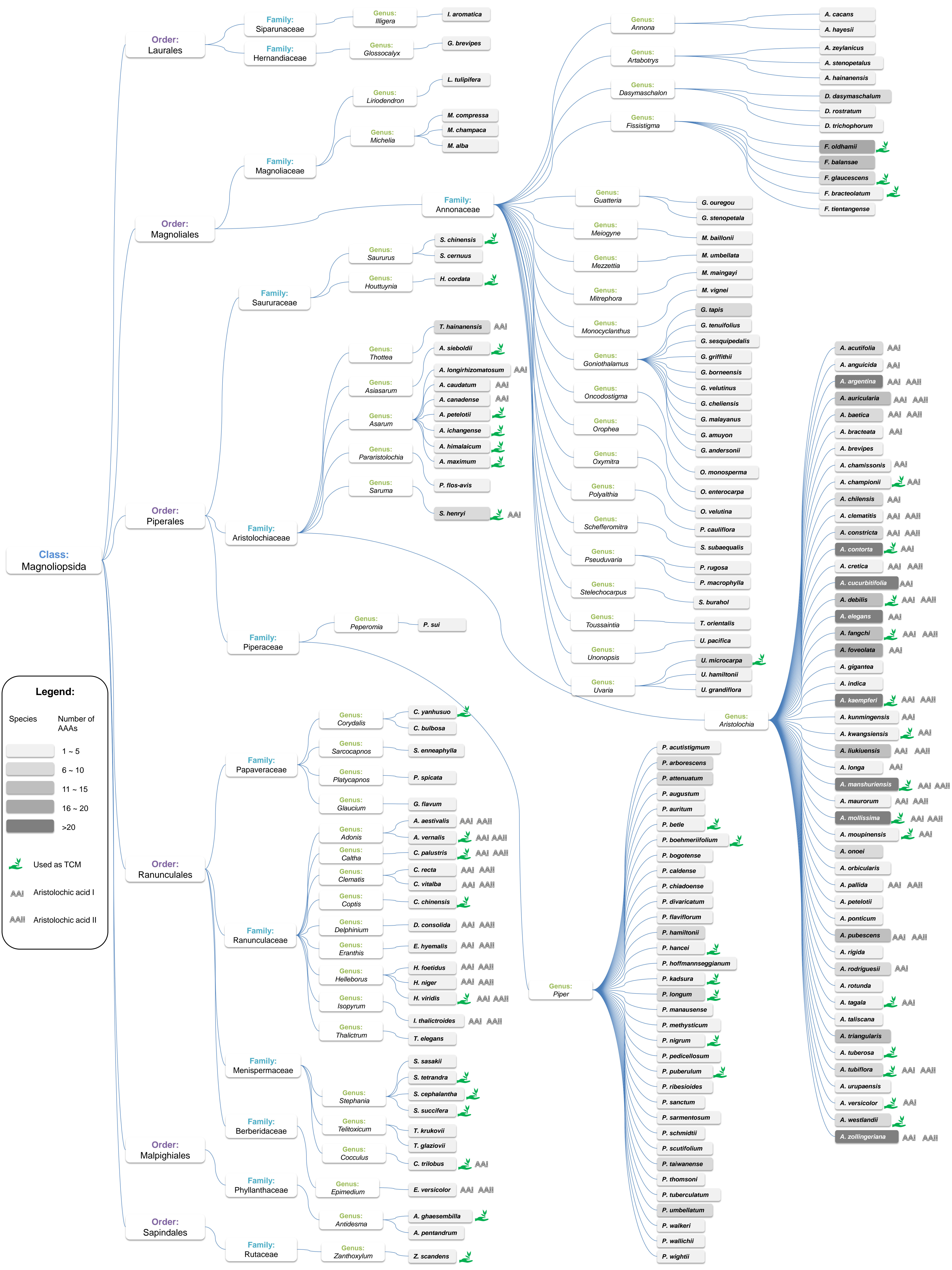

Supplement: Supplementary file 1 [file biomolecules-11-01344-s001.zip › Supplementary Materials/Supplementary Figures/Figure S3.pdf]

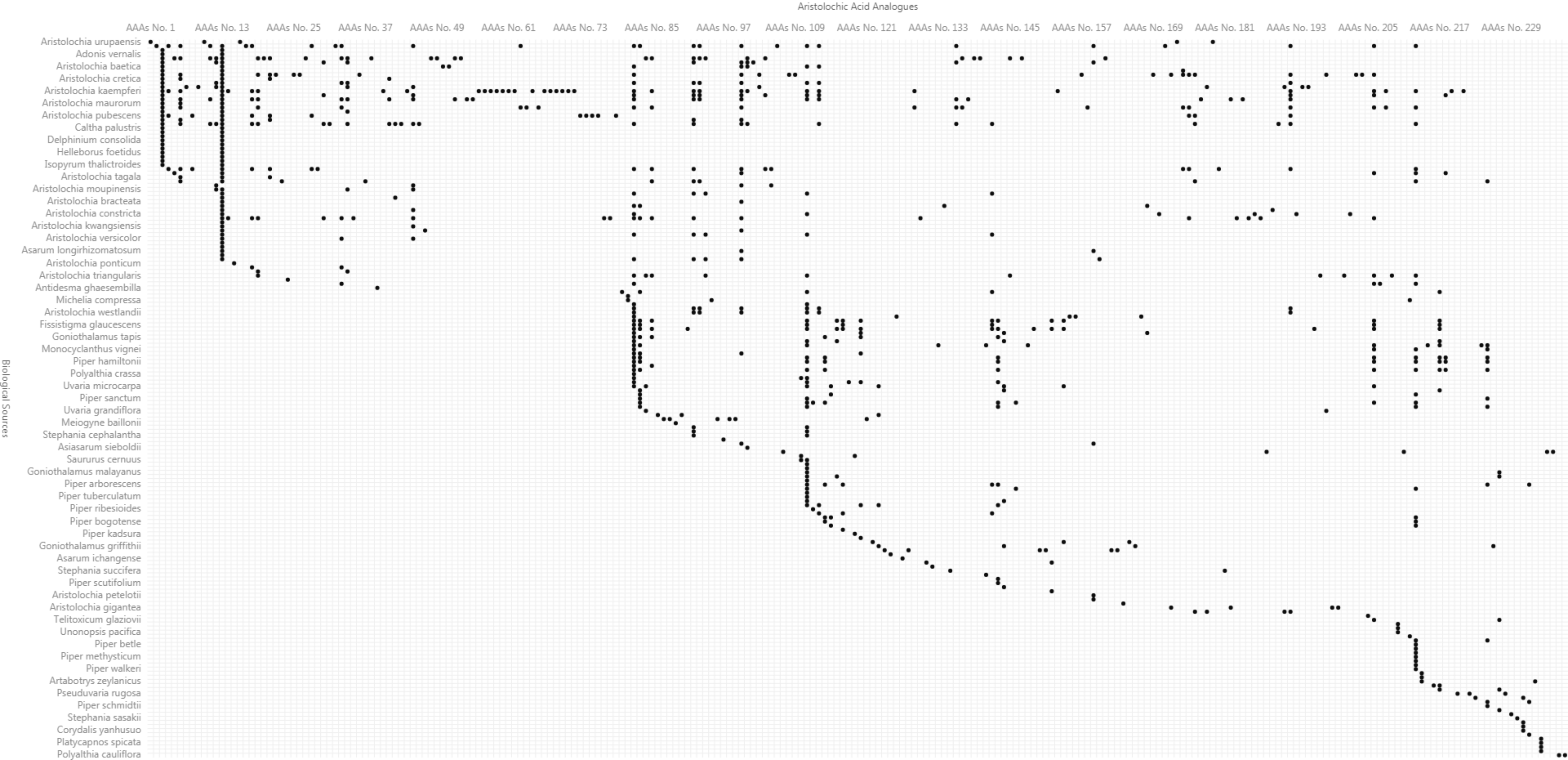

Supplement: Supplementary file 1 [file biomolecules-11-01344-s001.zip › Supplementary Materials/Supplementary Figures/Figure S4.pdf]

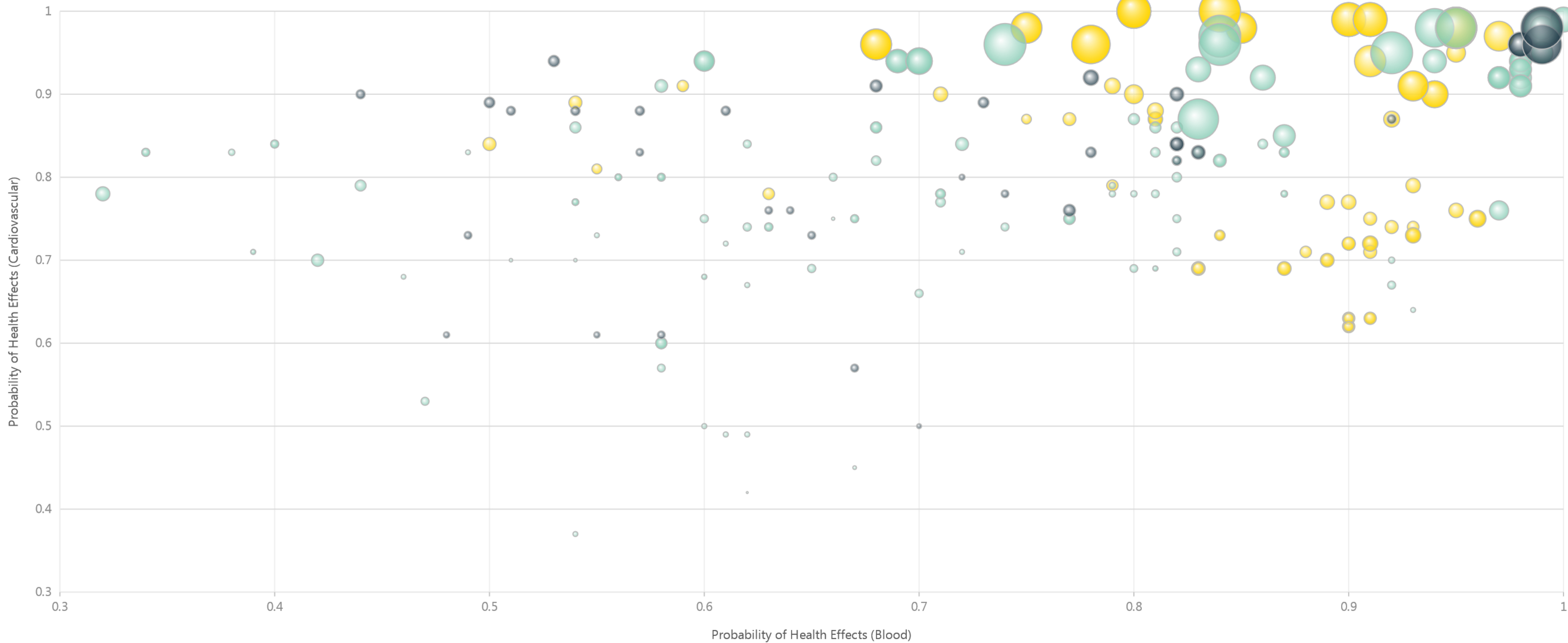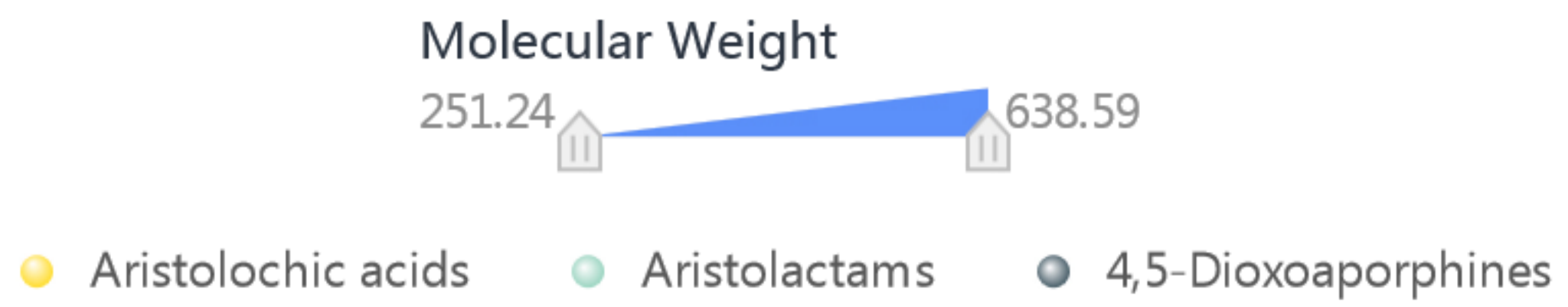

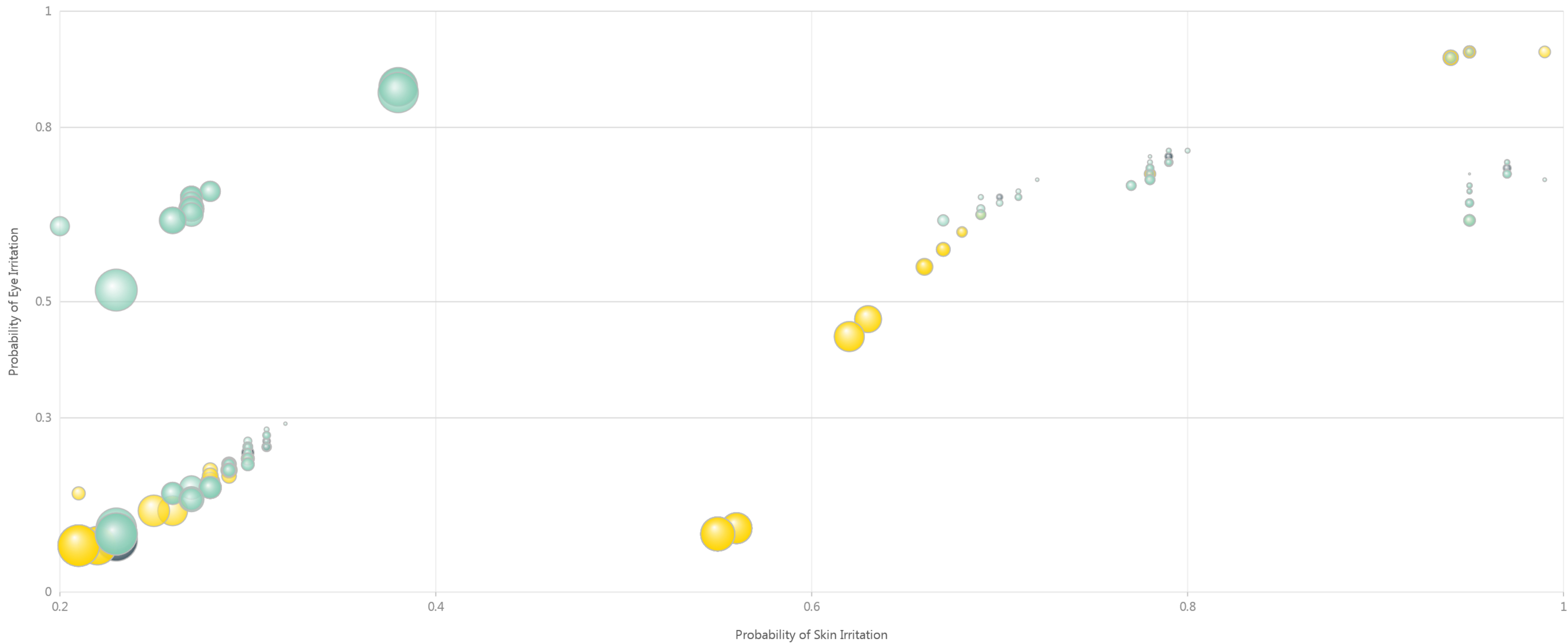

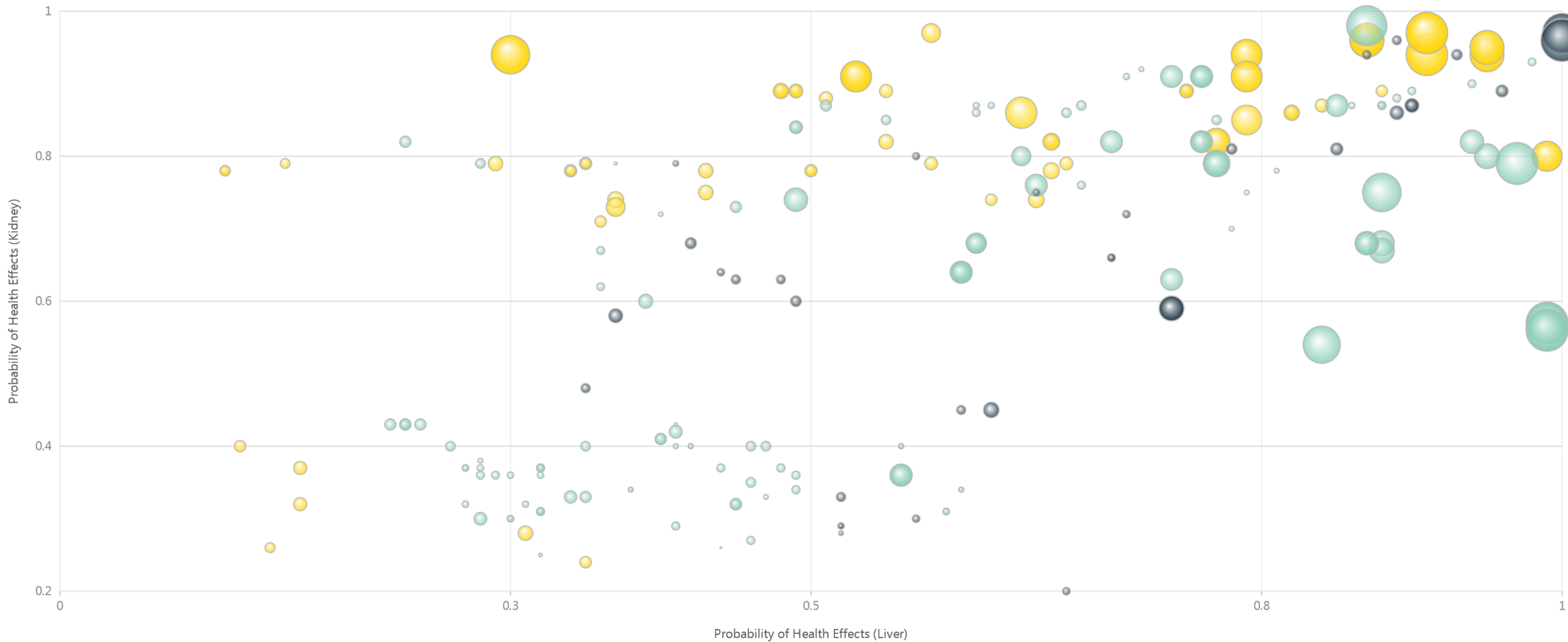

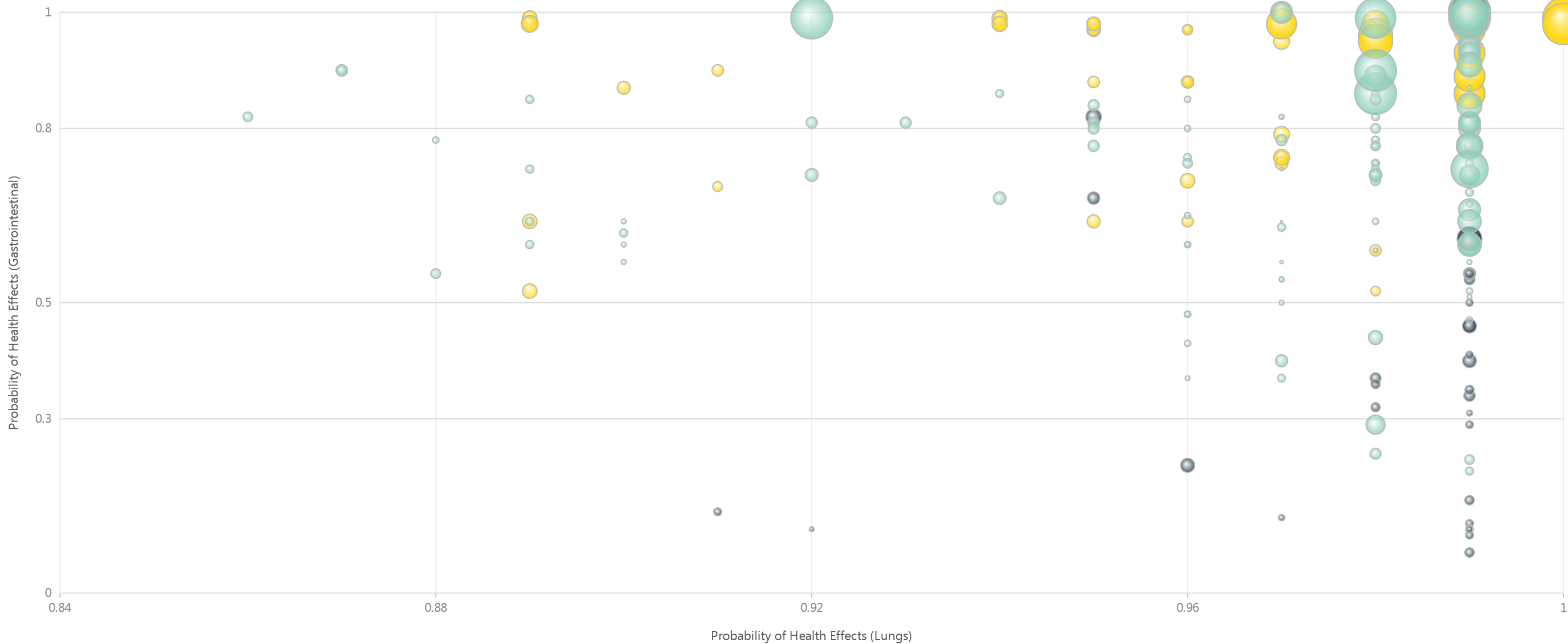

Supplement: Supplementary file 1 [file biomolecules-11-01344-s001.zip › Supplementary Materials/Supplementary Figures/Figure S5.pdf]

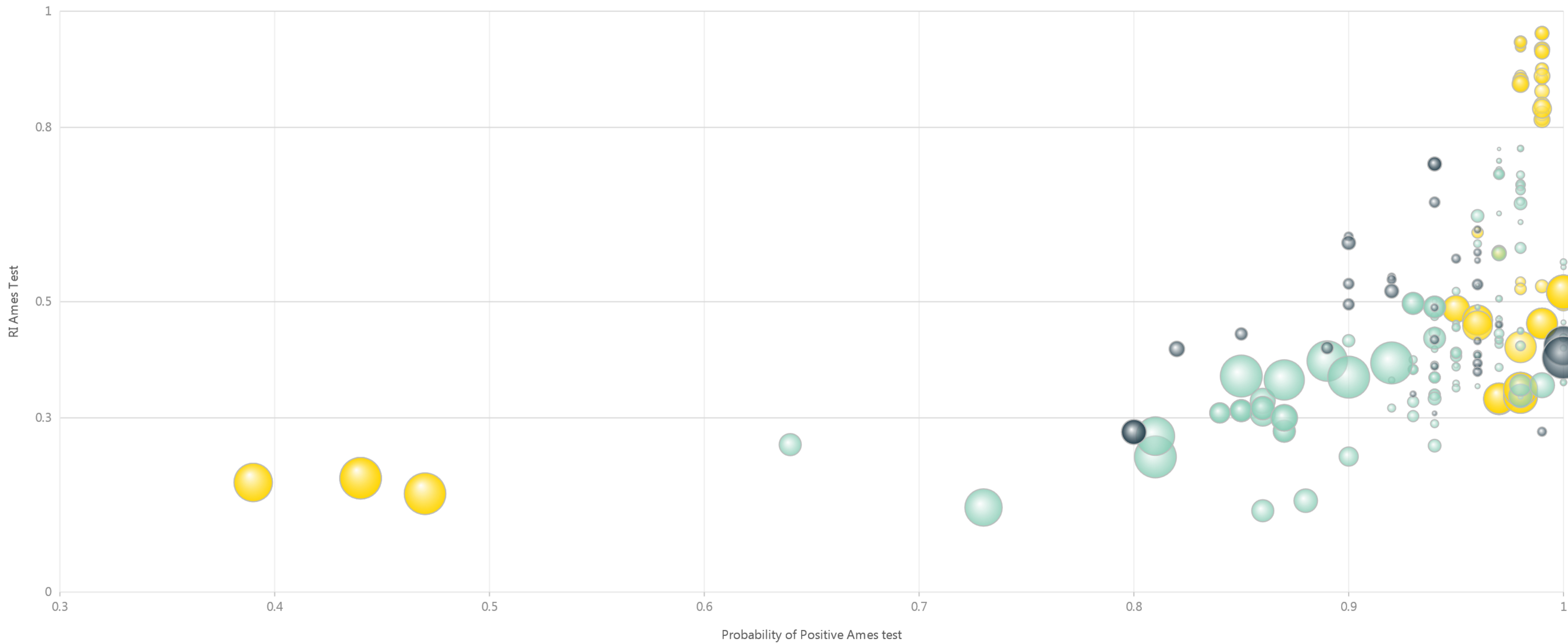



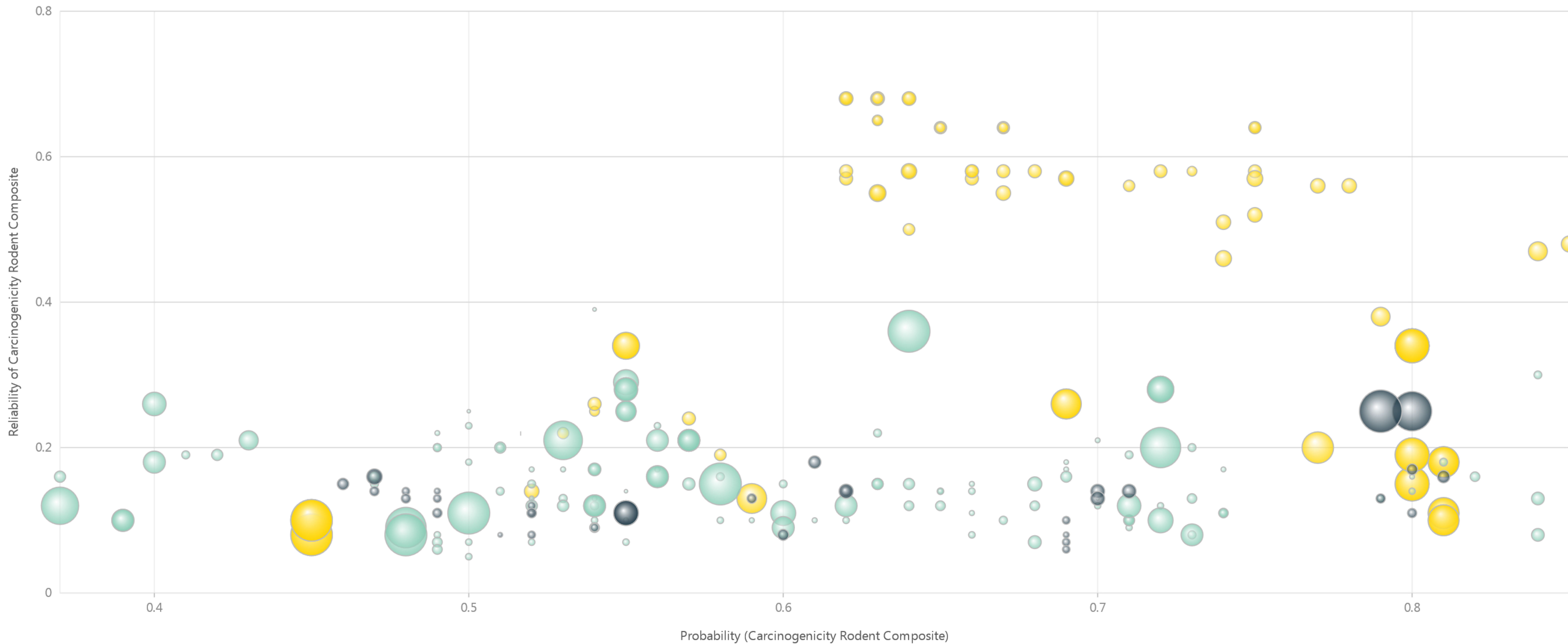

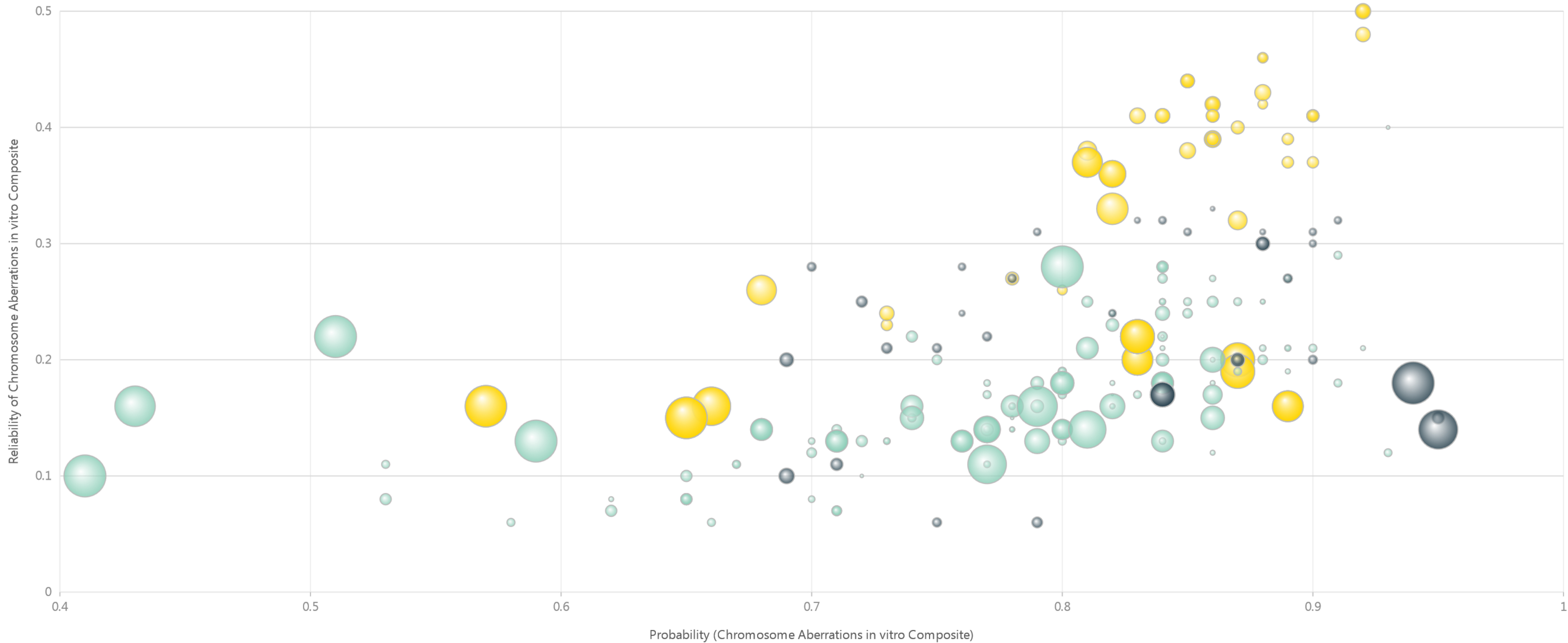

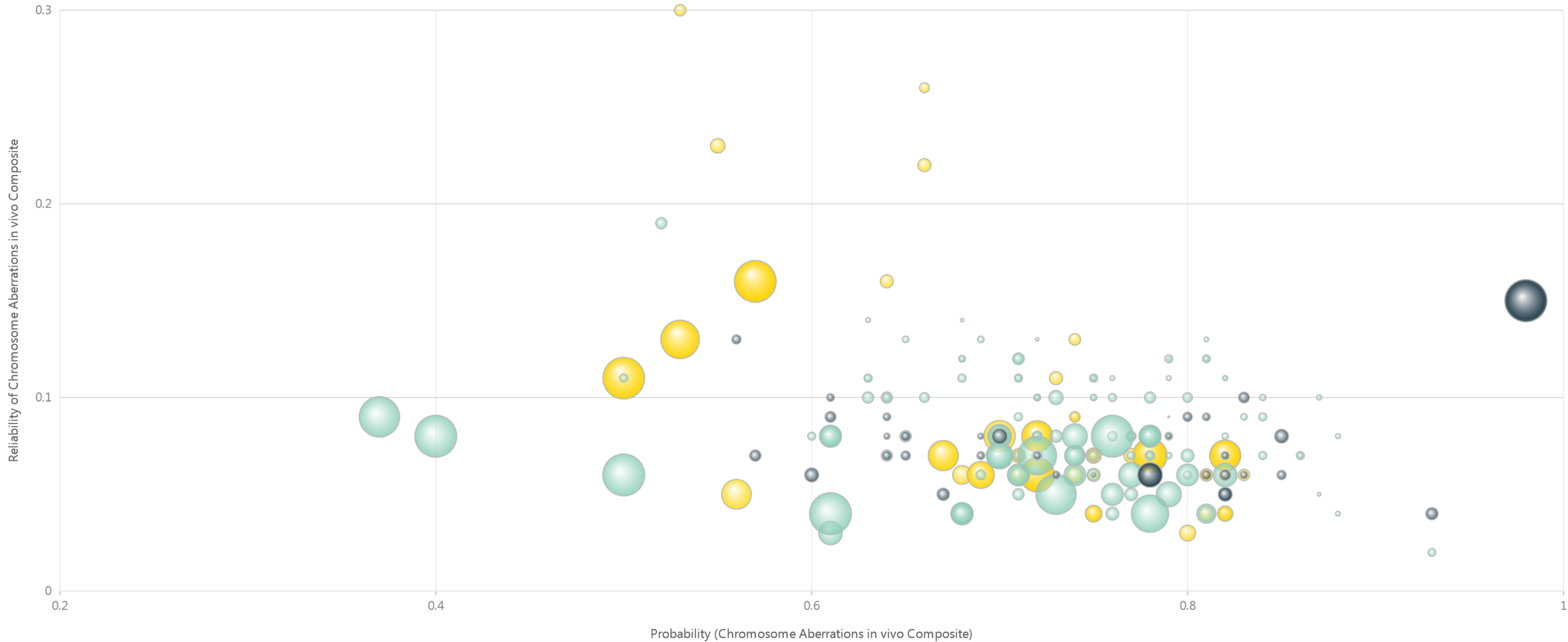

Supplement: Supplementary file 1 [file biomolecules-11-01344-s001.zip › Supplementary Materials/Supplementary Figures/Figure S7.pdf]
